# Equivalent Pore Channel Model for Fluid Flow in Rock Based on Microscale X-ray CT Imaging

**DOI:** 10.3390/ma13112619

**Published:** 2020-06-08

**Authors:** Chae-Soon Choi, Yong-Ki Lee, Jae-Joon Song

**Affiliations:** Department of Energy Resources Engineering, Research Institute of Energy and Resources, Seoul National University, Seoul 04750, Korea; ccsarchman@snu.ac.kr (C.-S.C.); yonggi12@snu.ac.kr (Y.-K.L.)

**Keywords:** sandstone, micro-CT image analysis, coupled pore channel model, permeability, direct numerical simulation

## Abstract

Pore-scale modeling with a reconstructed rock microstructure has become a dominant technique for fluid flow characterization in rock thanks to technological improvements in X-ray computed tomography (CT) imaging. A new method for the construction of a pore channel model from micro-CT image analysis is suggested to improve computational efficiency by simplifying a highly complex pore structure. Ternary segmentation was applied through matching a pore volume experimentally measured by mercury intrusion porosimetry with a CT image voxel volume to distinguish regions denoted as “apparent” and “indistinct” pores. The developed pore channel model, with distinct domains of different pore phases, captures the pore shape dependence of flow in two dimensions and a tortuous flow path in three dimensions. All factors determining these geometric characteristics were identified by CT image analysis. Computation of an interaction flow regime with apparent and indistinct pore domains was conducted using both the Stokes and Brinkman equations. The coupling was successfully simulated and evaluated against the experimental results of permeability derived from Darcy’s law. Reasonable agreement was found between the permeability derived from the pore channel model and that estimated experimentally. However, the model is still incapable of accurate flow modeling in very low-permeability rock. Direct numerical simulation in a computational domain with a complex pore space was also performed to compare its accuracy and efficiency with the pore channel model. Both schemes achieved reasonable results, but the pore channel model was more computationally efficient.

## 1. Introduction

An interpretation of fluid flow characteristics in porous rock is essential for many applications and engineering processes, such as enhanced oil recovery and carbon dioxide storage. The pore shape and size can now be accurately quantified [1,2] from microscale imaging technology like NMR and SEM. Many hydraulic properties have been estimated with consideration of the various features of porous rock characterized by complex and disordered pore shape. Therefore, many efforts have been made to properly acquire the micro-nano scale details of porous media. Based on these micro scale features of porous rock, pore-scale modeling with a reconstructed microstructure has become a dominant technique for fluid flow characterization. There are many computational methods like bundle of capillary tube modeling, direct pore scale modeling, and pore network modeling [3]. They differ in terms of their method to represent the pore space according to the geometry type, like bundled capillary, an extracted network of connected pores, and directly reconstructed 3D images. Among various modeling techniques, with the improved technology of X-ray computed tomography (hereafter micro-CT) imaging, some recent studies have focused on direct flow simulations with complex pore spaces obtained from micro-CT images. Through the pore geometry after being meshed, the flow characterization yield insights into the effect of pore morphology on both microscopic and macroscopic flow properties [4,5,6]. Key features of the fluid velocity and pressure field in mesh domain have been examined by the finite volume method [7,8] or finite element method [9]. In general, these direct pore-scale models should represent the microstructures of porous media and accurately reflect the original images. However, using a Cartesian grid derived from 3D binary CT images makes it hard to ensure the required mesh quality because of the complex pore geometry [10]. Such simulations also still impose computationally demanding requirements to accurately simulate the flow behavior [1,11].

To solve these difficulties in fluid flow characterization, the purpose of this paper is to suggest a technique for the construction of a pore channel model from micro-CT image analysis. The basis of this study is a generalized model of pressure-driven flow based on a steady state Hagen–Poiseuille flow in cylindrical capillaries. Many theoretical studies have analyzed imbibition and hydraulic resistance mechanisms through straight or tortuous channels with different geometrical properties [12,13,14,15]. This study aimed to extract a representative streamline channel in porous rock via ternary image segmentation to distinguish between “apparent” and “indistinct” pores. Micro-CT images were examined and a porous morphological structure was characterized using a commercial image processing program, Avizo. A technique for construction of the pore channel model was proposed. The model simultaneously represents the shape-dependent resistance of flow in each apparent and indistinct pore area and the effect of tortuous flow channels. The obtained domain was further converted into a finite element model using AutoCAD program for construction of 3D domain and COMSOL multiphysics for finite element numerical analysis. A simulation with the developed model was achieved and evaluated against experimental results in the Darcy flow regime of low flow velocity. Additionally, to examine the calculation efficiency and accuracy, a finite volume method to solve the Stokes equation was applied using a pore structural mesh with complex geometric details.

## 2. Specimen and Microscale X-ray CT Imaging

**Figure 1:**
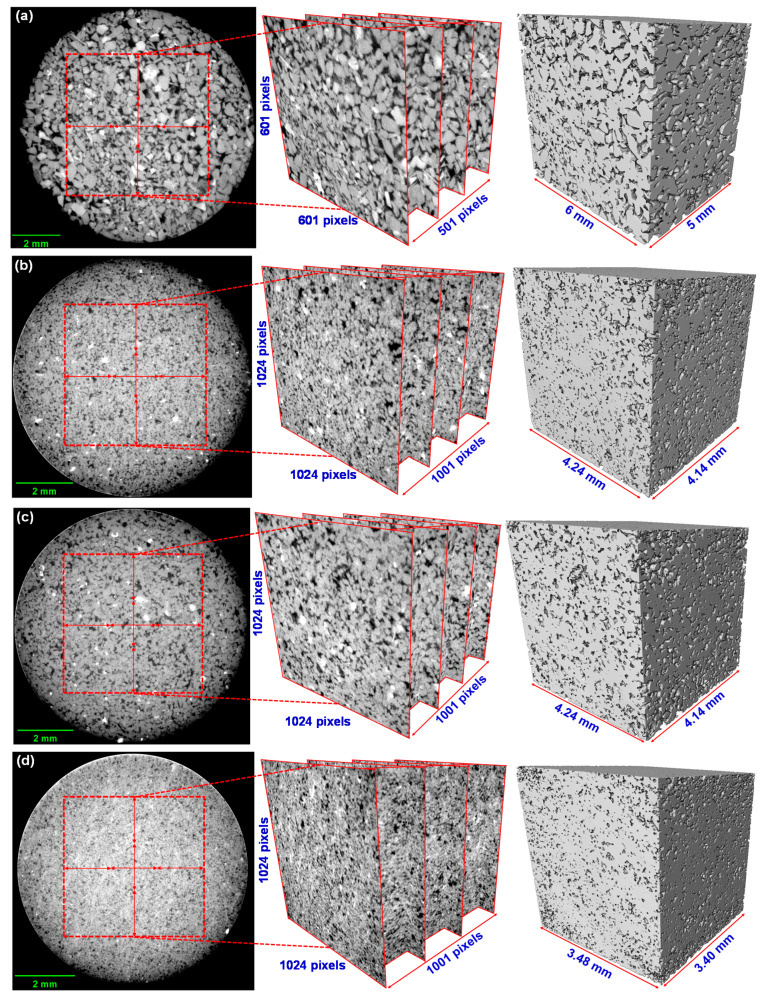

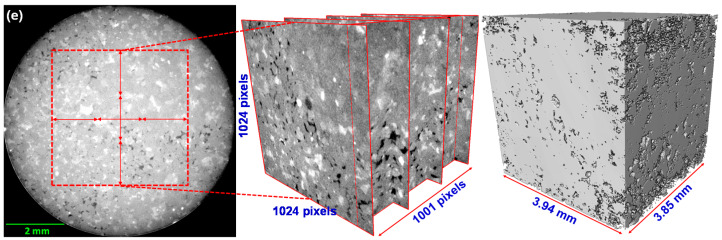
Typical 2D cross-sectional images of tested sandstones obtained by micro-CT imaging. In the resulting grayscale measurement plane, greater densities of attenuated pixels show brighter regions representing a dense solid. The regions are presented by 3D domains using a rendering process. (**a**) Boise sandstone (**b**) Berea sandstone (**c**) Buff Berea sandstone (**d**) Bandera sandstone (**e**) Linyi sandstone.

Cylindrical specimens of five kinds of sandstones (Boise, Buff Berea, Berea, Bandera, and Linyi) with diameters of 5–10 mm were prepared. The five sandstones are outcrops of subsurface formations in the United States or China. Imaging was performed using X-ray micro-CT equipment at the Korea Institute of Civil Engineering and Building Technology (KICT). The system allows a maximum of 320 kV radiation energy output and has a minimum focal spot size of 400 nm with a digital flat panel detector. Each sandstone specimen had already been characterized for hydraulic properties through a mercury intrusion porosimetry (MIP) test in this laboratory [16]. The measured pore median diameter and porosity are described in Table 1. For each sandstone sample, the MIP test was conducted three times to ensure reliability. A standard deviation is also shown in parentheses as well as an average value. Typically, the median diameter is used as a criterion related to the critical length for determining the voxel size in the resolution of micro-CT images. Very high resolution with a small voxel size can provide very precise pore geometries [17]. However, overly high resolution can lead to a long exposure time and limitations on the micro-CT device setting. A high computational demand is also inevitable for certain aspects of image processing when generating a very high-resolution image. Therefore, when determining an appropriate voxel size, the voxels should be smaller than the median pore diameter to ensure sufficiently reliable results and to facilitate qualitative and quantitative pore geometric information [18]. In this study, according to the median pore diameter from MIP test results of each sandstone, raw images were acquired with various resolutions leading to voxel sizes ranging from 3 to 10 μm, with parameters of 120 kV and 170–220 μA for the X-ray source. A representative element volume (REV) consisting of 1001 slices of 1024 × 1024 voxels (only 500 slices of 601 × 601 in the case of Boise sandstone) was extracted from each of the original micro-CT images and reconstructed 3D domains. The stacked image visualization, and analysis were performed using the commercial Avizo software. In Figure 1, the voxel number and domain length dependent on the resolution are indicated on the reconstructed REV domains.

## 3. Development of the Pore Channel Model

### 3.1. Ternary Segmentation

A segmentation or labeling process in the image analysis of rock is a critical step to separate the different phases and areas presented in raw micro-CT images [1,19,20]. The process also converts the attenuated images into a quantitative characterization of the pore geometry. The aim is to classify and assign the different phases corresponding to each voxel in a 3D image depending on the gray level, expressed as an intensity value. Many previous studies have obtained binary segmentation images after a pre-processing method, like denoising, and established a pore structure to implement a numerical simulation [21,22,23]. However, binary segmentation assigns each individual voxel to one of only two phases, solid or pore. Thus, an irregular pore geometry cannot be resolved accurately. Efforts to supplement binarization have been conducted by applying ternary thresholding methods [24,25,26,27]. Ternary segmentation is based on the determination of a threshold intensity value, which denotes whether each voxel corresponds to a solid particle, pore, or another phase. In a grayscale image, ternary segmentation defines a dark voxel with a low intensity value as an apparent pore, while a white voxel with a high intensity value is defined as a solid. Then, importantly, gray level voxels with an unclear assignment are defined as either a solid particle or pore to avoid an overestimation of pore space. Various methods for identifying a suitable threshold value have been attempted, like matching an experimentally measured porosity with the CT voxel volume, running a region-growing algorithm or performing image clustering from a bimodal graph of intensity values [25,28,29,30]. A pore size distribution (PSD) curve estimated by an MIP test can also be used for classifying those regions [27,31]. Kang et al. [27] proposed a new variable named the separating diameter, and developed a method of comparing the classified voxel volume with the pore fraction volume from the PSD curve. They assigned various separating diameters as multiple values of the unit voxel size depending on the resolution of micro-CT. Through the ternary segmentation of images using different separating diameters, permeability was directly estimated based on a lattice Boltzmann method. When the separating diameter was close to the median pore size, the computed permeability was similar to experimental results.

**Figure 2:**
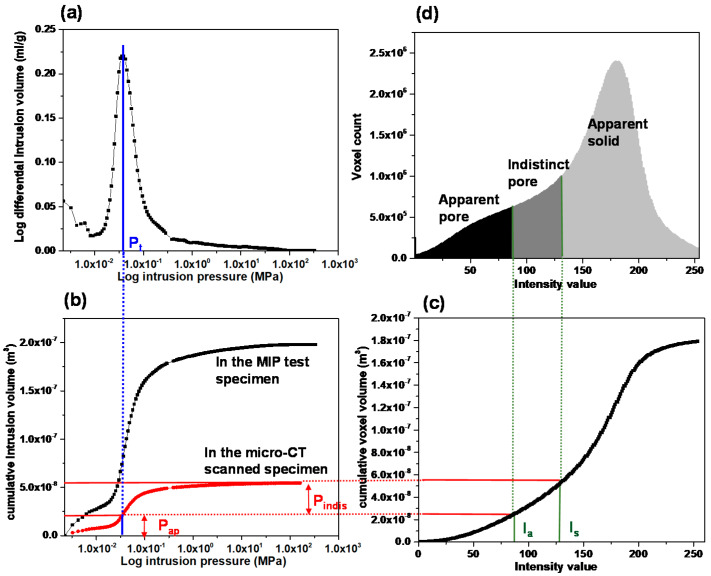
Segmentation and designation processes for three-phase materials consisting of apparent pores (*P_ap_*), indistinct pores (*P_indis_*) and apparent solid (*S_ap_*). Data were obtained from the MIP test (**a**,**b**) and the micro-CT image process on a case of Boise sandstone (**c**,**d**).

**Figure 3:**
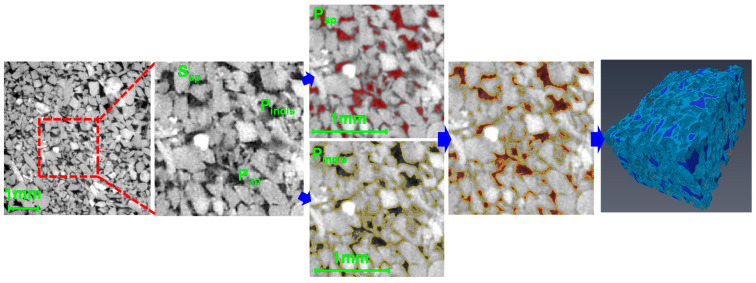
2D segmented section images for designation of apparent pores, indistinct pores and solid phase. Based on the proposed method, the voxels of each phase were accurately detected and a 3D domain containing each designated phase was finally obtained (in case of Boise sandstone).

This study proposed a ternary segmentation process that matches the pore volume data from MIP test and the CT image voxel volume. As mentioned in Section 2, the REV region had been assigned from the middle of the original 2D section image for excluding the uneven intensity value at the circular boundary. Only for the REV region, the three phases could be defined as (1) apparent pores (hereafter *P_ap_*) with a low intensity, (2) indistinct pores (*P_indis_*) with an intermediate intensity, and (3) apparent solid (*S_ap_*) with a high intensity. The threshold values for distinguishing the three phases were examined through a modified method based on that proposed by Kang et al. [27]. In the MIP test, the volume of intruded mercury was measured as a function of increasing intrusion pressure in several stages, as shown through a graph of log differential intruded mercury versus intrusion pressure (Figure 2a; Choi et al. [16]). The graph shows an inflection point, which is defined as the threshold pressure (*P_t_*). Katz and Thompson [32,33] proposed a permeability prediction method using the *P_t_* and a corresponding characteristic length (*L_t_*). Many previous studies have examined the validity of the proposed method and highlighted the importance of the *P_t_*, because the *L_t_* is a unique representative length scale for fluid flow and dominates the permeability [16,32,33,34]. With this representation of the *P_t_*, a criterion for deciding the threshold intensity value in micro-CT-images to distinguish between *P_ap_* and *P_indis_* was suggested in this study. After deciding *P_t_*, a cumulative intrusion pore volume per specimen mass was obtained from the MIP test, which can be directly converted to the pore volume of a micro-CT scanned specimen whose mass is known (Figure 2b). This approach is able to determine which voxels correspond to the *P_ap_* or *P_indis_* by counting voxels from the lowest intensity value until the voxel volume of *P_ap_* or *P_indis_* becomes the same as the cumulative intrusion volume from the MIP test (Figure 2c). Then, the intensity value, *I_a_*, for differentiation of the two types of pore can be determined by matching the volume data from the MIP test with the threshold pressure (*P_t_*) and the voxel volume in the reconstructed micro-CT image. Finally, all of the remaining voxels not assigned to the pore phases *P_ap_* and *P_indis_* are designated as *S_ap_* (Figure 2d). Figure 2 illustrates an application of Boise sandstone and this algorithm was also applied to the other sandstones to perform the ternary segmentation approach (please refer to Figures S1–S5 in the Supplementary Materials for each sandstone). Image processing techniques in AVIZO, including edge preserving to ensure clear contours of each phase and the removal of non-connected pores, were also carried out before the segmentation. Figure 3 shows the 2D section and reconstructed 3D domain, which indicate the classification of each phase. From the segmented 2D section, accurate extraction of the *P_ap_* and *P_indis_* can be confirmed by visible distinction between dark voxels and gray scale voxels. The intensity values used for separating the three phases of each sandstone are indicated in Table 2. In addition, the pore area and perimeter information of separated *P_ap_* and *P_indis_* can be estimated. Table 2 indicates the average value of these pore characteristic length from the numerous pores contained within each specimen.

### 3.2. Analysis and Determination of Representative Pore Shape

The pore shapes corresponding to the *P_ap_* and *P_indis_* must be obtained to determine the composition of the modeled pore channel. As mentioned in Section 1, the basis of the pressure-driven flow channel model was derived from Hagen–Poiseuille theory. The basis of the pressure-driven flow channel model was derived from Hagen–Poiseuille theory. The classical Hagen–Poiseuille equation for pressure-driven flow model is as follows:

(1) $$\frac{dP}{dx}=\frac{8\mu Q}{\pi r_{e}^{4}}$$

where *Q* is flow rate *μ* is the viscosity and *r_e_* is hydraulic radius. Based on the general Hagen–Poiseuille formula, previous studies have proposed various types of modified Hagen–Poiseuille formulas for considering a non-circular pore shape. The form of the equation varies greatly depending on a definition of length characteristics of the pores included in the equations. The flow resistance in flow channel is relative with the perimeter of cross section in contact with fluid. Therefore, a given pore shape is characterized by dimensionless compactness (*C*) expressed with its perimeter (*P*) and area (*A*):

(2) $$C=\frac{P^{2}(perimeter)}{A_{c}}$$

or hydraulic diameter (*D_h_*):

(3) $$D_{h}=\frac{4A}{P}$$

Based on these expressions for shape-dependence of the flow resistance, the pressure loss effect can be represented by a geometrical friction factor. The correction factor has been expressed in various forms in previous studies of the Hagen–Poiseuille equation. Especially, Mortensen et al. [35] proposed modified expression using geometrical correction factor (α) for various pore shapes, including rectangular, triangular, as well as ecliptic:

(4) $$\frac{dP}{dx}=\frac{\alpha \mu Q}{A^{2}}$$

Mortensen et al. [35] applied a shape perturbation theory to extend the analytical results of Hagen-Poiseuille flow by applying the compactness (*C*) to examine the correction factor (α). In the shape perturbation theory, the pore shape is described in parametric coordinates (*x*, *y*) defined by a transformation in Cartesian form:

(5) x=l[1+εsin(kθ)]cosθ

(6) y=l[1+εsin(kθ)]sinθ

where *k* is an integer larger than 2, defining the order of harmonic perturbation, and *l* is the length scale. A perturbation parameter, *ε*, is adopted to characterize the deformation of pore shape. For *ε* = 0, the shape is unperturbed. As *θ* is varied from 0 to 2π, the shape is transformed in a suitable way to formulate the perturbed Hagen-Poiseuille problem. Each parameter in the shape transformation is related to the pore geometry as follows:

(7) $$k=\sqrt{\frac{C-4\pi }{2\pi \varepsilon^{2}}+1}$$

(8) $$l=\sqrt{\frac{A}{(1+\frac{1}{2}\varepsilon^{2})\pi }}$$

**Figure 4:**
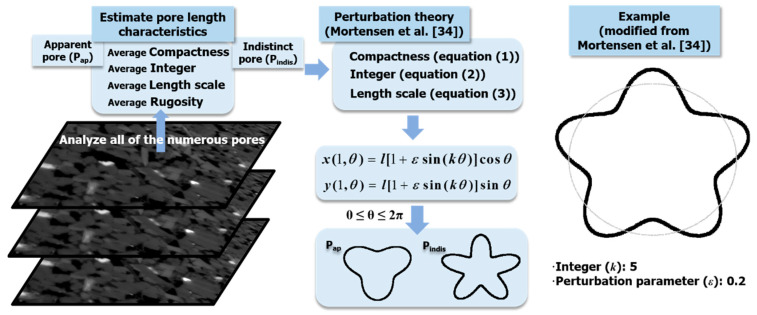
Flow chart to briefly describe the estimation process of representative pore geometry and example of perturbed cross-section.

In this study, quantitative results of the pore characteristics were obtained from the analysis of micro-CT images and it is confirmed that each specimen contained numerous pores through the segmentation process. Therefore, estimation of each parameter for adaption of the perturbation theory have been subjected to all pores. Their average values were used to extract one representative pore shape (as shown in Figure 4). Specifically, the important perturbation parameter (*ε*) was determined using average rugosity data to classify the contour of each pore as smooth or not. In the Avizo software, the labeling analysis contains a function for finding the rugosity. Similarly, with the other constants of *k* and *l*, the average values of the perturbation parameters (*ε*) were used to determine the representative pore shape. Table 3 shows the average value with standard deviation in parentheses and reconstruction results of a representative pore shape of each sandstone. The shape of *P_indis_* encloses *P_ap_* because it was confirmed through the 2D cross-sectional image analysis that the *P_indis_* pores surround the *P_ap_* pores (Figure 3). In the pore channel model, the length scale parameter (*l*) determines the area of each *P_ap_* and *P_indis_*. Therefore, the region around the black line in Table 3 is equal to the average area of the *P_ap_* in the micro-CT images, and the region around the red line minus the area of the black line is the actual average pore area of the *P_indis_*.

### 3.3. Application of Tortuous Flow Path

**Figure 5:**
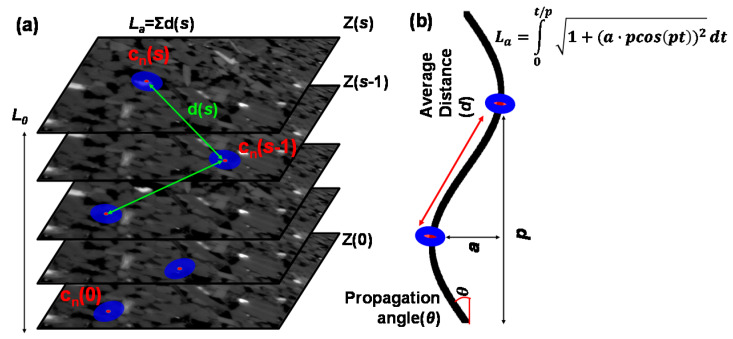
Method for construction of a tortuous flow channel model. (**a**) Estimation of centroid tortuosity in Avizo program. (**b**) Incorporation of a sine curve with information of connected pores along the *z*-axis into a tortuous flow channel.

Tortuosity is a key parameter for the investigation of fluid flow in a porous rock [35,36]. Normally, the tortuosity is defined as the ratio between the actual length of the fluid flow path through a porous medium and a corresponding straight distance, such as the specimen length. In Avizo, a centroid method can be used for estimation of the tortuosity through an investigation of connected pores. This module first searches for the centroid of the pore phase for each plane in the stacked images. Then, it computes the path length through the centroids (*L_a_* = Σd(*s*) in Figure 5a), and estimates the tortuosity through dividing *L_a_* by the number of planes, i.e., the *z*-axis voxel height of the stacked image (*L*_0_). In this study, as shown in Figure 5b, it is assumed that the length of a sine curve with a specific amplitude (*a*) and period (*p*) is equal to the actual path length of the connected pores. Therefore, through an integral form for the length of the sine curve, the pore channel model expresses the tortuous flow path as follows:

(9) $$L_{a}=\int_{0}^{t/p}\sqrt{1+{\left(a\cdot p\cos pt\right)}^{2}}dt$$

The determination of amplitude and period is important to ensure the reasonability of the tortuous path model. We utilize the image analysis results to assign an appropriate amplitude and period of the sine curve. In Avizo, the average distance (*d*) and the propagation angle (*θ*) of connected pores along the *z*-axis can also be estimated through the 3D length in the length orientation module. As shown in Figure 5b, the amplitude and period can be derived from the distance and angle. The results in Table 4 indicate the tortuosity of the flow paths of each sandstone. The tortuosity, average distance and propagation angle of the connected pores can be designated as tortuosity factors. The factors that determine the 3D tortuous geometry were estimated based on the total pore phase area calculated by summing the *P_ap_* and *P_indis_*. The 2D cross-sections of the *P_ap_* and *P_indis_* with the representative shapes have the same channel along the tortuous flow path. In all of the *z*-axis voxel heights, the indicated straight distance (*L*_0_) has a value of about 0.1 mm (resolution × number of stack images, i.e., 0.004138 mm × 25 voxels in the case of Berea sandstone).

### 3.4. Construction of 3D Domain and Its Properties

**Figure 6:**
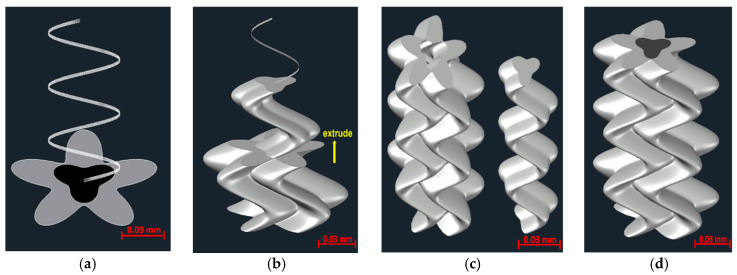
Process for construction of a 3D domain with a separated pore and a tortuous flow channel in case of Berea sandstone. (**a**) 2D surface each feature of pore shape and 3D spline of tortuous flow path; (**b**) extrusion of the surface along the *z*-axis spline; (**c**) two separated domains with *P_ap_* and *P_indis_*; (**d**) coupled flow regime with the two domains.

Finally, we produced a distinct 3D domain containing the calculated data for *P_ap_* and *P_indis_* through a combination of 2D sections with the representative pore shapes and a 3D tortuous flow path. First, the cloud point data for each feature of pore shape and tortuous flow path were imported into the AutoCAD program. Multiple points were connected with a spline and converted into a 2D surface and 3D spline (Figure 6a). Then, the surface was extruded along the *z*-axis spline expressed by the sine curve (Figure 6b). The *P_ap_* and *P_indis_* surface objects were individually converted into a 3D solid domain through the extrusion process. A part of the *P_ap_* included in the *P_indis_* domain was extracted to separate the two domains (Figure 6c). The two divided domains were coupled and treated with different physical equations under a coupling calculation to realize the bulk permeability of one pore channel (Figure 6d). In the commercial program COMSOL Multiphysics, a coupled flow regime with the two domains was easily meshed and set up. Both the Stokes and Brinkman equations were solved to conduct a fluid flow simulation in the pore channel model. Some assumptions were made with respect to the creeping flow, neglecting inertial effects. The Stokes equation was applied in the free flow region of the *P_ap_*, while the partially permeable region of the *P_indis_* was governed by the Brinkman equation. The system consisted of two domains with one fluid phase, and a coupling between free flow in the *P_ap_* and Darcy-like flow in the *P_indis_* was implemented. The free flow in *P_ap_* was described by the stationary, incompressible Stokes equations:

(10) $$\begin{array}{l} 0=-\nabla p+\nabla \cdot \mu (\nabla u+\nabla u^{T})\\ \nabla \cdot u=0 \end{array}$$

while in the *P_indis_*, the Brinkman equation described the flow:

(11) $$\begin{array}{l} 0=-\nabla p+\nabla \cdot \frac{\mu }{\phi_{indis}}(\nabla u+\nabla u^{T})-\frac{\mu }{K_{indis}}u\\ \nabla \cdot u=0 \end{array}$$

where *u* refers to the velocity in the domain (m/s), *μ* denotes the dynamic viscosity (Pa·s), and *p* is the fluid pressure (Pa). In the Brinkman equation, the key macroscopic parameters of *K* and *ϕ* denote permeability (m^2^) and porosity (dimensionless). Therefore, appropriate input parameters for the *P_indis_* should be determined and assigned. In this study, the image analysis data of the pore volume and hydraulic diameter shown in Section 3 were adopted to obtain the porosity and permeability. The porosity of *P_indis_* could be easily derived from the pore volume data from the micro-CT voxels. The *P_ap_* are complete pores (porosity: 1), hence the porosity of *P_indis_* could be calculated by dividing their volume (*V_indis_*) by the remainder of the total micro-CT image volume (*V_total_*) after subtracting the volume of *P_ap_* (*V_ap_*)

(12) $$\phi_{indis}=\frac{V_{indis}}{V_{total}-V_{ap}}$$

To determine the permeability of *P_indis_*, the Kozeny–Carman equation was adopted, which relates the permeability of pores to their geometry. Kozeny and Carman modeled a porous medium composed of a bundle of parallel channels with parameters of spherical particle diameter (*d_s_*), tortuosity (*L_a_*/*L*_0_) and a relation between superficial velocity (*v_s_*) and interstitial (pore or actual) velocity (*v_s_*/*ϕ*):

(13) $$\frac{\Delta P}{L}=\frac{72\mu v_{s}L_{a}{\left(1-\phi \right)}^{2}}{L_{0}d_{s}^{2}\phi^{3}}=\frac{180\mu v_{s}{\left(1-\phi \right)}^{2}}{d_{s}^{2}\phi^{3}}$$

In the equation, the Kozeny constant, given a value of 180, is dependent on an assumed factor related to the tortuous flow channel length and the shape of the particle. In many instances, the constant is determined under an assumption of cylindrical pores or spherical particles. This means that the shape factor is 6 and the fixed tortuosity about 2.5. In this study, to avoid these restrictive assumptions, we adopted a form of the Kozeny–Carman equation expressed only by the hydraulic radius for utilizing the pore geometry information had already been acquired through image processing. Through a comparison with Darcy’s law, the Kozeny–Carman equation can be expressed as simply an alternative analytical expression for the permeability, as below:

(14) $$\frac{2\mu v_{s}L_{a}}{r_{h}^{2}\phi L_{0}}=\frac{\mu v_{s}}{K}$$

Because the effect of tortuosity (*L_a_*/*L*_0_) had already been included in the model geometry, expressed by a sinusoidal curve (see Table 4), only the hydraulic radius (*r_h_*) and porosity (*ϕ*) of the *P_indis_* phase were used to derive the permeability. Table 5 shows the permeability and porosity values assigned to the *P_indis_* domain.

## 4. Numerical Modeling and Results

**Figure 7:**
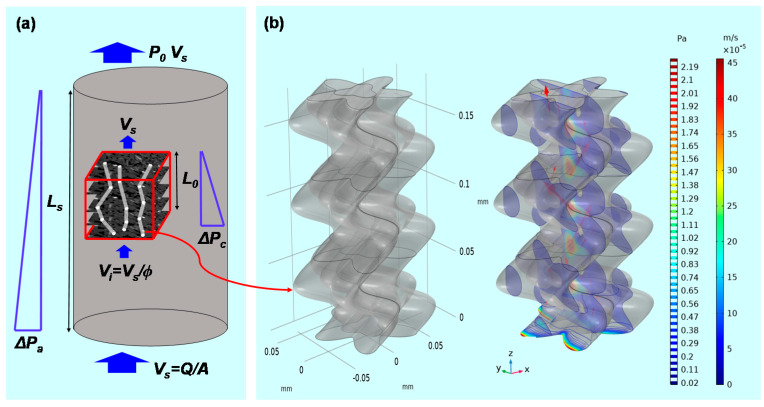
Depiction of fluid pressure and velocity field through a porous specimen and consideration of a micro-CT image volume fraction. (**a**) Illustration of the experimental condition of steady state flow under a constant pressure gradient. Boundary notation explains the parameters for adopting Darcy’s law in both the experiment and numerical simulation. (**b**) Computed pressure and velocity field in the pore channel model of Berea sandstone.

For a numerical simulation using the developed pore channel model, an appropriate boundary condition must be assigned to distinguish the pore-scale forces used in the Stokes–Brinkman equation from the core-scale fluid pressure drop found in Darcy flow. A core flooding test had previously been conducted in this laboratory using supercritical CO_2_ and the five types of sandstone (Choi & Song [16]). Through several flow tests, the permeability of each sandstone had been deduced experimentally. To simulate the CO_2_ flooding test, simulation parameters corresponding to the experimental conditions should be imposed on the pore channel model. Table 6 lists the input parameters. For matching the experimental conditions, the CO_2_ properties are assumed to be constant across the pore channel model under a temperature of 50 °C and pressure of 10 MPa. Figure 7 compares the core flooding experiment and the numerical simulation based on the developed pore channel model with a brief description (also refer to Figure S6 in Supplementary Materials). The flow rate through the specimen is *Q* (m^3^/s) and the cross-sectional area is *A* (m^2^), thus a general superficial or Darcy velocity *v_s_* is easily calculated as *Q* divided by *A*. In accordance with the core flooding test, the specimen area had a constant value of 2.25 × 10^−3^ m^2^ and the flow rate was assumed to be a fixed value of 1.7 × 10^−8^ m^3^/s to simulate a laminar flow. Therefore, the superficial velocity is a constant value of 7.29 × 10^−6^ m/s. The upstream condition induced a resistance to fluid flow and gave rise to a pressure increase at the bottom side. Therefore, the permeability of the core specimen and the micro-CT volume fraction could be estimated through the induced pressure gradient along the specimen length of *L_s_* and by considering the distance *L_o_*, which is the voxel height. As mentioned in Section 3.3, the *z*-axis straight distance (*L*_0_) on all specimens has a value of about 0.1 mm. It should be emphasized that this study considered only one representative pore channel from the micro-CT volume, which had the form of a pore channel bundle. The actual fluid velocity in the connected pores was calculated by considering the existence of a pore throat within the specimen, which accelerated the superficial velocity to preserve fluid continuity. Therefore, the interstitial velocity (actual or pore velocity) in the pore throat simply represents the increased velocity determined by the relation between the superficial velocity and the porosity of the specimen, as illustrated in Figure 7 [37,38,39,40]. In the developed model of a pore channel extracted from the void fraction of the micro-CT volume, the applied inlet flow should take the interstitial velocity at the bottom side of the pore channel. Therefore, each different inlet velocity was assigned according to the porosity of the specimens, as listed in Table 6. After that, as shown in Figure 7, the permeability of the micro-CT volume fraction was calculated from the pressure gradient (Δ*P_c_*) along the micro-CT image voxel height (*L_o_*) and the outlet superficial velocity (*v_s_*):

(15) $$K=\frac{\mu v_{s}L_{o}}{\Delta P_{c}}$$

To obtain the average pressure gradient for a bundle of pore channels, the induced pressure must be integrated over the pore channels of different sizes and then divided by the number of channels [12,41]. However, it was assumed in this study that all connected pore channels were the same size as the representative pore channel, so the pressure gradient calculated from the numerical simulation could be directly taken as the macroscopic value for use with Darcy’s law. The permeability computed from the velocity and pressure field was compared with results of laboratory experiments (please refer to Tables S1–S6 in the Supplementary Materials for estimated values for Darcy’s law in pore channel model of each sandstones). The permeability measured through the experiments is mentioned in Choi & Song [16] as the average value. As shown in Table 7, the results exhibited satisfactory agreement in the relatively highly permeable rocks, with errors of less than 25 percent with respect to the experimental results. However, in the case of Bandera and Linyi sandstone, the discrepancy was found to be more than 50 percent. In the aggregate, these results indicate that the permeability obtained from the pore channel model was underestimated for the highly permeable rocks, while it was overestimated in the case of sandstone with low permeability. In this regard, previous studies of a pore capillary tube model noted a discrepancy with regard to a lattice-type network model, caused by not considering the interaction between adjacent tubes. Therefore, in the case of highly permeable rocks, it is proposed that the permeability was underestimated due to the neglect of interaction, as our numerical modeling method likewise used only one representative pore channel domain despite the existence of multiple pore channels. Meanwhile, the overestimated permeability of low-permeability rock is proposed to have been caused by a resolution problem of the micro-CT imaging. The resolution must be sufficient to identify the pore geometry in the case of a dense rock containing a small range of pore sizes. In the case of a low-permeability rock, the possibility of the presence of smaller pores below the resolution increases, which can cause a pore detection problem. Therefore, in this study, insufficient resolution for characterization of the *P_indis_* pore size of Bandera and Linyi sandstone might have led to the incorrect permeability of *P_indis_*, which was estimated through the Kozeny–Carman equation using the hydraulic radius. Therefore, it is assumed that the permeability was overestimated in the pore channel model coupled with the *P_ap_* and *P_indis_* domains. The next section will discuss how to improve the pore channel model and will compare it with a direct numerical simulation, i.e., a modeling approach using a finite volume mesh to preserve the complexity of pore space.

## 5. Discussion

### 5.1. Effect of Tortuosity Factor

As mentioned in Section 3.3, the tortuosity factors, including the tortuosity, average distance, and propagation angle of the connected pores, were determined through an investigation of the total pore phase (*P_ap_* + *P_indis_*) for each plane in the stacked image. However, the numerical simulation results using the tortuous flow path with these tortuosity factors had some disagreement with the experimental results. An improvement of the developed pore channel model can be expected by implementing the concept of preferential flow paths. Previous studies showed that fluid flow is most likely to occur along certain preferential flow paths formed in a porous medium [42,43,44]. Therefore, the spatial pressure distribution of the pore channels with a large hydraulic radius presumably controlled the induced flow velocity and pressure gradient in the specimen. Typically, in a micro- pore structure, the maximum flow velocity appears at the center of the apparent pores where a fully developed fluid flow occurs [45]. To simulate the preferential flow path effect, the tortuosity factors were derived by applying image processing only to *P_ap_*. Table 8 presents the tortuosity factors and sinusoidal flow paths in respect of *P_ap_*. A comparison with Table 4 indicates that the factors differ according to whether the total pore phase or only the *P_ap_* phase is considered. Overall, the average *z*-axis distance between connected pores and the tortuosity are found to increase. A reconstruction of the pore channel model applying the modified tortuosity factors was attempted. The same construction method described in Section 3 was adopted. The reconstructed 3D computational domain was used in the COMSOL program and the permeability was estimated through the fluid velocity and pressure field. Table 9 shows a simulation results of pressure and velocity fields in case of Boise sandstone (please refer to Tables S1–S6 in the Supplementary Materials for other sandstones). As shown in Table 10, a reasonable agreement was found between the permeability derived from the modified pore channel model and the experimentally estimated permeability. The modified model showed a much improved error rate compared with the pore channel model expressed using the tortuosity factors derived from the total pore area. However, the model is still currently incapable of accurate flow modeling for a very low-permeability rock like Linyi sandstone. In view of the lack of micro-CT resolution as mentioned in Section 4, the error is an artifact of the considerable simplification of the pore geometry by disregarding undetectable pores. A further improvement might be achieved by using high-resolution imaging techniques with sufficient computational capacity for construction of the connected pore space from the nanometer scale upwards.

### 5.2. Comparison with Direct Numerical Simulation

**Figure 8:**
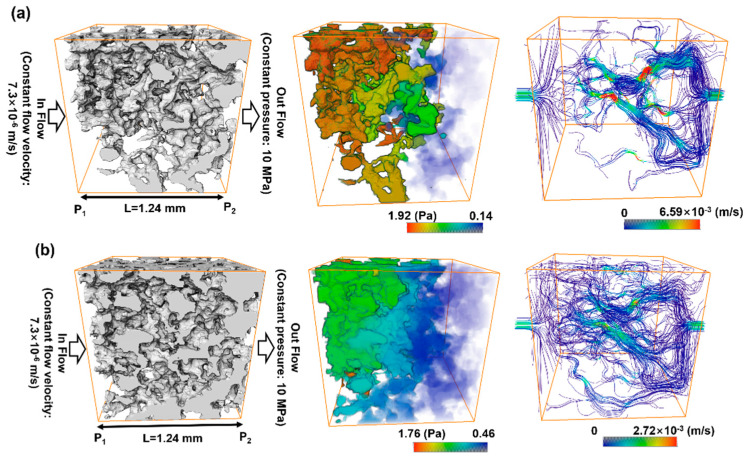
Computed velocity and pressure gradient field dependent on the pore structure in region of interest configured as (**a**) the *P_ap_* phase or (**b**) the *P_ap_* + *P_indis_* phase for Berea sandstone.

The resulting pore geometric description from the image processing could also be utilized for a direct numerical simulation (DNS). Several previous studies have highlighted the importance of DNS at the pore scale for better understanding of fluid flow physics, and DNS has been applied to validate a macroscopic model of porous media [25,46,47]. We compared the developed pore channel model with DNS in terms of their accuracy and efficiency. In DNS, the velocity and pressure field of the Stokes equation are calculated on a computational mesh representing complex pore spaces based on a discrete approximation. In this study, the simulation was performed using Avizo’s XLab module for simulating an absolute permeability experiment based on a finite volume method [48]. To compute a flow field driven by a pressure gradient, it is necessary that the key connected pore structure is identified and modeled to simplify the simulation. The principal pore space being meshed is also dependent on the threshold intensity value of the micro-CT image because the bulk volume size of the pore space is determined by deciding whether or not each voxel is a pore. Thus, in this study, the simulation case was classified using one of two criteria: the pore space was regarded as either the *P_ap_* phase only or as the total pore phase combining the *P_ap_* and *P_indis_* phases. Table 11 shows a significant difference in the key features of pore structure of the two cases. Note that a region of interest (ROI) from the micro-CT image voxel volume must be precisely chosen for representing the pore complexity and connectivity in DNS. The ROI volume influences the computational efficiency because a large volume will make the simulation very time-consuming. The results themselves are also dependent on the chosen ROI. An appropriate choice of ROI containing a connected pore structure was crucial to the permeability simulation. In the ROI domains, unnecessary fractions such as unconnected pore space, designated as island pores, were also found. The *z*-axis propagation process had the aim of detecting a main flow pathway through the ROI in shape of regular hexahedron. Therefore, the key pore space connected along the *z*-axis was effectively skeletonized after the island pores were classified as negligible. In the figures accompanying Table 11, the separated pores marked in red were deleted and then the velocity and pressure field was imposed across the remaining subvolume by solving the Stokes equation. Figure 8 indicates the results in the case of Berea sandstone (please refer to Figures S7–S11 in the Supplementary Materials for other sandstones). The application of Darcy’s law yields the permeability with fluid velocity and pressure gradient. The fluid viscosity and boundary condition is same with the COMSOL simulation of the pore channel model. As shown in Table 12, reasonable results were acquired when the simulation was performed on the pore structure configured only for the *P_ap_*. In the case of the computational domain built from the *P_ap_* + *P_indis_* phase, an overestimation of large connected pores was caused by considering the Pindis in the pore structure as a fully developed flow regime. Therefore, it is suggested that a pore structure constructed solely from *P_ap_* should be chosen for DNS. As mentioned in Section 5.1, an importance of the preferential flow path is recognized for constructing the pore geometry and understanding the fluid flow behavior in a porous medium. The predicted permeabilities from both DNS and the developed pore channel model indicated reasonable results. However, in DNS, if the computational domain was chosen with a 4 mm domain length, which was the target area for construction of the pore channel model as shown in Figure 2, it required a considerable working time of more than six hours and a large computational memory. These difficulties have also been encountered in previous studies [1,8]. It was therefore necessary to designate the ROI as a sufficiently small area (less than 2 mm^2^ in this study), which in turn led to a problem of dependence on the chosen region. This dependence was found to be critical in low-permeability rock like Bandera and Linyi sandstone. In fact, when several repeated DNS were conducted by specifying different ROIs, a significant deviation of estimated permeability, more than 2.96 × 10^−^^14^ m^2^, was found in Linyi and Bandera sandstone. The results of DNS in Table 12 show that the pore channel model most closely reproduced the experimental results. Therefore, both in terms of computational efficiency and dependence on the ROI, the pore channel model is somewhat advantageous for flow simulation.

## 6. Conclusions

In direct numerical simulation of pore-scale fluid flow, it can be difficult to ensure the required mesh quality because of the complexity of pore geometry. Additionally, the region of interest must be precisely chosen for the sake of computational efficiency and because of the dependence of the results on the choice of region. To overcome these difficulties, we developed a technique for the construction of a simple pore channel model from a micro-CT image analysis of various sandstones. The process can be briefly described as follows:

1. Representative streamline channels of five types of sandstones were determined from a ternary image segmentation to distinguish apparent and indistinct pores. Threshold intensity values of the micro-CT images were examined through matching experimentally measured pore volumes from MIP tests with the CT image voxel volumes.
2. In two dimensions, a shape perturbation theory was applied to extend the pore channel flow for the case of irregular pores with a shape-dependent flow resistance. The results of micro-CT image analysis of pore perimeter, area, and rugosity were used for determining the parameters in the perturbation theory. A representative pore shape for each of the different sandstones could be derived.
3. In three dimensions, the effect of tortuosity was modeled by expressing the flow as sinusoidal curves expressing the degree of tortuosity, average distance and propagation angle of connected pores along the *z*-axis. Each factor was also investigated through image analysis, and the results indicated a dependence on the chosen object region, i.e., whether the pore phase was defined as only the apparent pores or the combination of apparent and indistinct pores.
4. Distinct 3D domains of apparent and indistinct pores were constructed through combining a 2D section with representative pore shapes and a 3D tortuous flow path. Both the Stokes and Brinkman equations were solved to compute the interaction flow regime with the two domains. A coupling simulation was achieved and evaluated against the experimental results in the Darcy flow regime.

A reasonable agreement was found between the permeability derived from the pore channel model and the experimentally estimated permeability. The pore channel model expressed using tortuosity factors derived only from the apparent pore area showed much improvement in terms of permeability estimation. We also compared the developed pore channel model with a direct numerical simulation for the validation of its accuracy and efficiency. The permeabilities predicted from both the direct numerical simulation and the developed pore channel model indicated reasonable results. However, both in terms of computational efficiency and dependence on the region of interest, the developed pore channel model was distinctly advantageous. The developed pore channel model may provide a method to simplify complex pore geometries and prove suitable for fluid flow problems at the pore scale.

## Figures and Tables

**Table 1:** Results of mercury intrusion porosimetry test and voxel sizes of micro-CT images.

Pore Characteristics	Boise	Berea	Buff Berea	Bandera	Linyi
Average mean pore diameter (μm)	2.61 (±0.31)	0.75 (±0.03)	1.00 (±0.04)	0.24 (±0.01)	0.13 (±0.01)
Average median pore diameter (µm)	43.39 (±2.99)	11.69 (±0.18)	17.76 (±0.59)	5.03 (±1.30)	7.01 (±0.60)
Average porosity	29.5 (±0.76)	21.1 (±0.35)	23.8 (±0.71)	20.7 (±0.82)	10.1 (±0.44)
Voxel size (µm)	10	4.138	4.138	3.396	3.847

**Table 2:** Intensity values of the ternary segmentation and pore geometry data derived from image processing for characterization of apparent and indistinct pores.

Pore Phase	Title	Boise	Berea	Buff Berea	Bandera	Linyi
Intensity value	I_a_	87	85	97	87	107
	I_s_	131	128	143	118	121
Apparent pore	Average pore area (m^2^)	1.17 × 10^−8^ (± 2.41 × 10^−9^)	1.87 × 10^−9^ (±0.31 × 10^−9^)	3.81 × 10^−9^ (±0.67 × 10^−9^)	1.16 × 10^−9^ (±0.21 × 10^−9^)	1.96 × 10^−9^ (±0.57 × 10^−9^)
	Average Perimeter (μm)	433 (±55.8)	159 (±15.1)	239 (±26.3)	129 (±14.4)	152 (±25.2)
	Average Hydraulic D (μm)	66.4 (±4.17)	31.7 (±1.87)	41.2 (±2.54)	24.1 (±1.38)	26.2 (±2.05)
Indistinct pore	Average pore area (m^2^)	1.63 × 10^−8^ (±4.01 × 10^−9^)	3.04 × 10^−9^ (±0.50 × 10^−9^)	4.56 × 10^−9^ (±0.91 × 10^−9^)	1.35 × 10^−9^ (±0.27 × 10^−9^)	1.18 × 10^−9^ (±0.51 × 10^−9^)
	Average Perimeter (μm)	1341 (±316.2)	380 (±55.9)	597 (±112.1)	273 (±60.3)	153 (±44.4)
	Average Hydraulic D (μm)	42.8 (±1.57)	27.7 (±1.06)	28.2 (±1.06)	13.9 (±5.51)	13.9 (±8.88)

**Table 3:** Representative pore shape derived for each type of sandstone.

Boise	Berea	Buff Berea	Bandera	Linyi
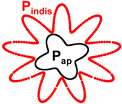	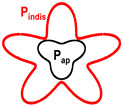	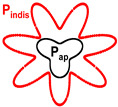	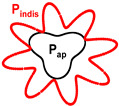	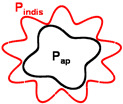
<*P_ap_*>	<*P_ap_*>	<*P_ap_*>	<*P_ap_*>	<*P_ap_*>
*C* = 21.1 (±1.3)	*C* = 17.7 (±0.7)	*C* = 19.8 (±1.0)	*C* = 18.3 (±0.9)	*C* = 18.0 (±1.2)
*k* = 4 (±2)	*k* = 3 (±0.3)	*k* = 3 (±0.4)	*k* = 3 (±0.4)	*k* = 4 (±0.5)
*l* = 4.51 (±0.38) × 10^−5^	*l* = 1.93 (±0.14) × 10^−5^	*l* = 2.64 (±0.21) × 10^−5^	*l* = 1.50 (±0.13) × 10^−5^	*l* =1.69 (±0.17) × 10^−5^
*ε* = 0.31 (±0.106)	*ε* = 0.30 (±0.095)	*ε* = 0.37 (±0.084)	*ε* = 0.27 (±0.094)	*ε* = 0.22 (±0.084)
<*P_indis_*>	<*P_indis_*>	<*P_indis_*>	<*P_indis_*>	<*P_indis_*>
*C* = 115.2 (±25.7)	*C* = 49.79 (±6.8)	*C* = 83.2 (±14.6)	*C* = 75.7 (±14.2)	*C* = 35.3 (±5.4)
*k* = 9 (±3)	*k* = 5 (±1.5)	*k* = 7 (±2.3)	*k* = 8 (±2.4)	*k* = 9 (±2.2)
*l* = 6.99 (±0.48) × 10^−5^	*l* = 3.00 (±0.17) × 10^−5^	*l* = 3.67 (±0.23) × 10^−5^	*l* = 2.02 (±0.11) × 10^−5^	*l* = 1.92 (±0.13) × 10^−5^
*ε* = 0.36 (±0.101)	*ε* = 0.38 (±0.084)	*ε* = 0.39 (±0.077)	*ε* = 0.32 (±0.097)	*ε* = 0.19 (±0.086)

**Table 4:** Tortuosity of each sandstone and tortuous flow paths expressed by a sine curve.

Tortuous Characteristics	Boise	Berea	Buff Berea	Bandera	Linyi
Tortuosity	1.44	1.99	1.77	1.72	2.75
Tortuous propagation angle (°)	47.4 (±2.6)	61.2 (±1.2)	63.8 (±6.7)	58.0 (±0.4)	48.7 (±2.5)
Avg. *z*-axis distance of connected pore (mm)	0.1135 (±1.41 × 10^−4^)	0.06034 (±6.55 × 10^−6^)	0.05518 (±9.14 × 10^−6^)	0.04635 (±2.71 × 10^−6^)	0.0529 (±5.29 × 10^−6^)
Tortuous flow path	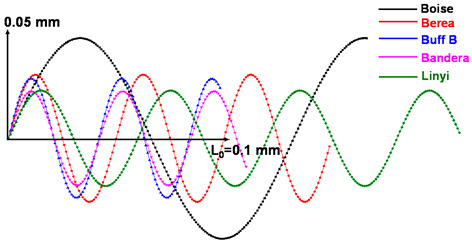				

**Table 5:** Macroscopic parameters of Brinkman equation for the *P_indis_* domain properties.

Indistinct Pore Properties	Boise	Berea	Buff Berea	Bandera	Linyi
Derived permeability (m^2^)	1.06 × 10^−11^	3.49 × 10^−12^	3.52 × 10^−12^	8.49 × 10^−13^	3.75 × 10^−13^
Porosity	0.186	0.147	0.157	0.124	0.063

**Table 6:** Numerical input data corresponding to core flooding experiment using CO_2_.

Property	Value		Description
*μ*	2.842 × 10^−5^ Pa·s		Dynamic viscosity
*v_s_*	7.29 × 10^−6^ m/s		Superficial velocity
*v_i_*	Boise	2.51 × 10^−5^ m/s	Interstitial inlet velocity
	Berea	3.46 × 10^−5^ m/s	
	Buff Berea	3.08 × 10^−5^ m/s	
	Bandera	3.56 × 10^−5^ m/s	
	Linyi	7.35 × 10^−5^ m/s	
*P*_0_	10 MPa		Initial outlet pressure

**Table 7:** Comparison of modeled and experimental permeability of each sandstone.

Results		Boise	Berea	Buff Berea	Bandera	Linyi
Permeability (m^2^)	Pore channel model	9.49 × 10^−13^	1.04 × 10^−13^	2.17 × 10^−13^	5.01 × 10^−14^	1.80 × 10^−14^
	Experiment	1.14 × 10^−12^ (±1.08 × 10^−13^)	1.34 × 10^−13^ (±4.32 × 10^−15^)	2.78 × 10^−13^ (±1.81 × 10^−14^)	3.30 × 10^−14^ (±1.06 × 10^−15^)	2.47 × 10^−15^ (±8.06 × 10^−17^)

**Table 8:** Tortuosity factors determined solely from the apparent pore phase of each sandstone.

Tortuous Characteristics	Boise	Berea	Buff Berea	Bandera	Linyi
Tortuosity	1.93	3.59	2.54	2.70	3.96
Tortuous propagation angle (°)	44.7 (±0.7)	56.4 (±0.3)	57.7 (±0.6)	58.3 (±0.2)	51.4 (±2.3)
Avg. *z*-axis distance of connected pores (mm)	0.1613 (±0.49 × 10^−4^)	0.08935 (±0.99 × 10^−5^)	0.07961 (±0.12 × 10^−4^)	0.05961 (±0.52 × 10^−5^)	0.0598 (±0.68 × 10^−5^)
Tortuous flow path	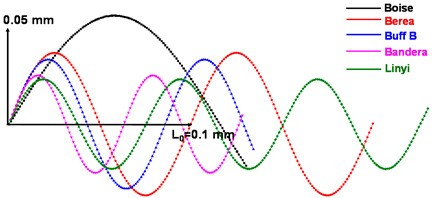				

**Table 9:** Computational results of fluid pressure and velocity field in the pore channel model of Boise sandstone. The tortuosity, average distance and propagation angle of connected pores of (**a**) the total pore phase and (**b**) the apparent pore phase were applied to the 3D flow path geometry.

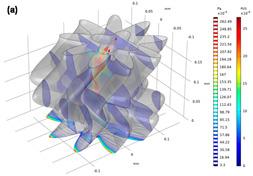	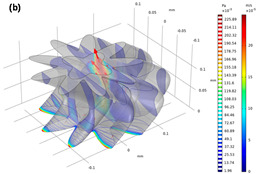
Average outlet superficial velocity (*v_s_*, 10^−6^ m/s): 7.185	Average outlet superficial velocity (*v_s_*, 10^−6^ m/s): 7.196
Average pressure gradient (Δ*P_c_*, Pa): 0.0216	Average pressure gradient (Δ*P_c_*, Pa): 0.0179

**Table 10:** Simulation results of permeability from the modified pore channel model using the tortuosity factors derived from applying image processing only to the apparent pore phase.

Results		Boise	Berea	Buff Berea	Bandera	Linyi
Permeability (m^2^)	Modified pore channel model	1.14 × 10^−12^	1.29 × 10^−13^	2.75 × 10^−13^	3.41 × 10^−14^	1.20 × 10^−14^
	Experiment	1.14 × 10^−12^ (±1.08 × 10^−13^)	1.34 × 10^−13^ (±4.32 × 10^−15^)	2.78 × 10^−13^ (±1.81 × 10^−14^)	3.30 × 10^−14^ (±1.06 × 10^−15^)	2.47 × 10^−15^ (±8.06 × 10^−17^)

**Table 11:** Variability of connected pore structure domain according to criteria determining which phases to include in the pore space of Berea sandstone. After a region of interest was extracted with a size of 201~301 tetrahedral voxels, unconnected pores at each domain were designated as negligible regions.

Object Phase	A *z*-axis Connected Pore Structure	Extract A Region of Interest	Detection of Unconnected Pores
Apparent pore (*P_ap_*)	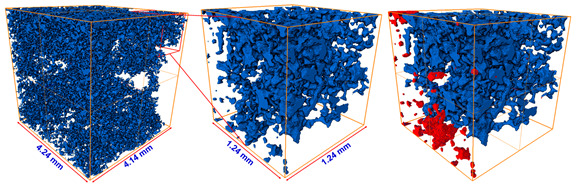		
Total pore (*P_ap_* + *P_indis_*)	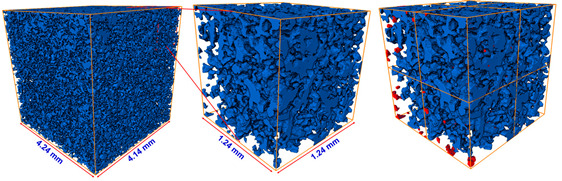		

**Table 12:** Direct numerical simulation results of two pore structures having different geometry according to the criterion for defining the pore phase.

Results	Object Phase		Boise	Berea	Buff Berea	Bandera	Linyi
Permeability (m^2^)	Direct numerical simulation	Apparent pore (*P_ap_*)	1.01 × 10^−12^	1.45 × 10^−^^13^	2.92 × 10^−^^13^	3.04 × 10^−^^14^	6.22 × 10^−^^15^
		Total pore (*P_ap_* + *P_indis_*)	1.26 × 10^−^^12^	1.98 × 10^−^^13^	3.74 × 10^−^^13^	5.60 × 10^−^^14^	3.15 × 10^−^^14^
	Pore channel model		1.14 × 10^−^^12^	1.29 × 10^−^^13^	2.75 × 10^−^^13^	3.41 × 10^−^^14^	1.20 × 10^−^^14^
	Experiment		1.14 × 10^−^^12^	1.34 × 10^−^^13^	2.78 × 10^−^^13^	3.30 × 10^−^^14^	2.47 × 10^−^^15^

## Supplementary Materials

The following are available online at https://www.mdpi.com/1996-1944/13/11/2619/s1, Figure S1: Segmentation and designation processes for three-phase materials consisting of apparent pores (*P_ap_*), indistinct pores (*P_indis_*) and apparent solid (*S_ap_*). Data were obtained from the MIP test (a,b) and the micro-CT image process on a case of Boise sandstone (c,d), Figure S2: Segmentation and designation processes for three-phase materials consisting of apparent pores (*P_ap_*), indistinct pores (*P_indis_*) and apparent solid (*S_ap_*). Data were obtained from the MIP test (a,b) and the micro-CT image process on a case of Berea sandstone (c,d), Figure S3: Segmentation and designation processes for three-phase materials consisting of apparent pores (*P_ap_*), indistinct pores (*P_indis_*) and apparent solid (*S_ap_*). Data were obtained from the MIP test (a,b) and the micro-CT image process on a case of Buff Berea sandstone (c,d), Figure S4: Segmentation and designation processes for three-phase materials consisting of apparent pores (*P_ap_*), indistinct pores (*P_indis_*) and apparent solid (*S_ap_*). Data were obtained from the MIP test (a,b) and the micro-CT image process on a case of Bandera sandstone (c,d), Figure S5: Segmentation and designation processes for three-phase materials consisting of apparent pores (*P_ap_*), indistinct pores (*P_indis_*) and apparent solid (*S_ap_*). Data were obtained from the MIP test (a,b) and the micro-CT image process on a case of Linyi sandstone (c,d), Figure S6: Depiction of fluid pressure and velocity field through a porous specimen and consideration of a micro-CT image volume fraction. Illustration of the experimental condition of steady state flow under a constant pressure gradient. Boundary notation explains the parameters for adopting Darcy’s law in both the experiment and numerical simulation, Figure S7: Computed velocity and pressure gradient field dependent on the pore structure in region of interest configured as (a) the *P_ap_* phase or (b) the *P_ap_* + *P_indis_* phase for Boise sandstone, Figure S8: Computed velocity and pressure gradient field dependent on the pore structure in region of interest configured as (a) the *P_ap_* phase or (b) the *P_ap_* + *P_indis_* phase for Berea sandstone, Figure S9: Computed velocity and pressure gradient field dependent on the pore structure in region of interest configured as (a) the *P_ap_* phase or (b) the *P_ap_* + *P_indis_* phase for Buff Berea sandstone, Figure S10: Computed velocity and pressure gradient field dependent on the pore structure in region of interest configured as (a) the *P_ap_* phase or (b) the *P_ap_* + *P_indis_* phase for Bandera sandstone, Figure S11: Computed velocity and pressure gradient field dependent on the pore structure in region of interest configured as (a) the *P_ap_* phase or (b) the *P_ap_* + *P_indis_* phase for Linyi sandstone; Table S1: Numerical input data corresponding to core flooding experiment using CO_2_, Table S2: Computational results of fluid pressure and velocity field in the pore channel model of Boise sandstone, Table S3: Computational results of fluid pressure and velocity field in the pore channel model of Berea sandstone, Table S4: Computational results of fluid pressure and velocity field in the pore channel model of Buff Berea sandstone, Table S5: Computational results of fluid pressure and velocity field in the pore channel model of Bandera sandstone, Table S6: Computational results of fluid pressure and velocity field in the pore channel model of Linyi sandstone.
