# Supplementary material for: Equivalent Pore Channel Model for Fluid Flow in Rock Based on Microscale X-ray CT Imaging
Source: Materials (Basel). 2020 Jun 8;13(11):2619. doi: 10.3390/ma13112619 (PMC7321608; doi:10.3390/ma13112619)
Supplement: Supplementary file 1 [file materials-13-02619-s001.pdf]

materials

Supplementary Materials

Equivalent Pore Channel Model for Fluid Flow in Rock Based on Microscale X-ray CT Imaging

Chae-Soon Choi, Yong-Ki Lee and Jae-Joon Song *

Department of Energy Resources Engineering, Research Institute of Energy and Resources, Seoul National University, Seoul 04750, Korea; ccsarchman@snu.ac.kr (C.-S.C.); yonggi12@snu.ac.kr (Y.-K.L.)

* Correspondence: songjj@snu.ac.kr

Received: 9 May 2020; Accepted: 1 June 2020; Published: date

Contents of This File

Tables S1–S6

Figures S1–S11

Introduction

This study aimed to extract a representative streamline channel in porous rock via ternary image segmentation to distinguish between “apparent” and “indistinct” pores. A ternary segmentation process was proposed to match a threshold pressure measured by the MIP test to the CT image voxel volume. In the MIP test, the volume of intruded mercury was measured as a function of increasing intrusion pressure in several stages, as shown through a graph of log differential intruded mercury versus intrusion pressure (graph (a) of Figures S1–S5). The graph shows an inflection point, which is defined as the threshold pressure (P_t). Katz and Thompson [32,33] proposed a permeability prediction method using the P_t and a corresponding characteristic length (L_t). Many previous studies have examined the validity of the proposed method and highlighted the importance of the P_t, because the L_t is a unique representative length scale for fluid flow and dominates the permeability [16,32–34]. With this representation of the P_t, a criterion for deciding the threshold intensity value in micro-CT-images to distinguish between P_{ap} and P_{indis} was suggested in this study. After deciding P_t, a cumulative intrusion pore volume per specimen mass was obtained from the MIP test, which can be directly converted to the pore volume of a micro-CT scanned specimen whose mass is known (graph (b) of Figures S1–S5). This approach is able to determine which voxels correspond to the P_{ap} or P_{indis} by counting voxels from the lowest intensity value until the voxel volume of P_{ap} or P_{indis} becomes the same as the cumulative intrusion volume from the MIP test (graph (c) of Figures S1–S5). Then, the intensity value, I_a, for differentiation of the two types of pore can be determined by matching the volume data from the MIP test with the threshold pressure (P_t) and the voxel volume in the reconstructed micro-CT image. Finally, all of the remaining voxels not assigned to the pore phases P_{ap} and P_{indis} are designated as S_{ap} (graph (d) of Figures S1–S5).

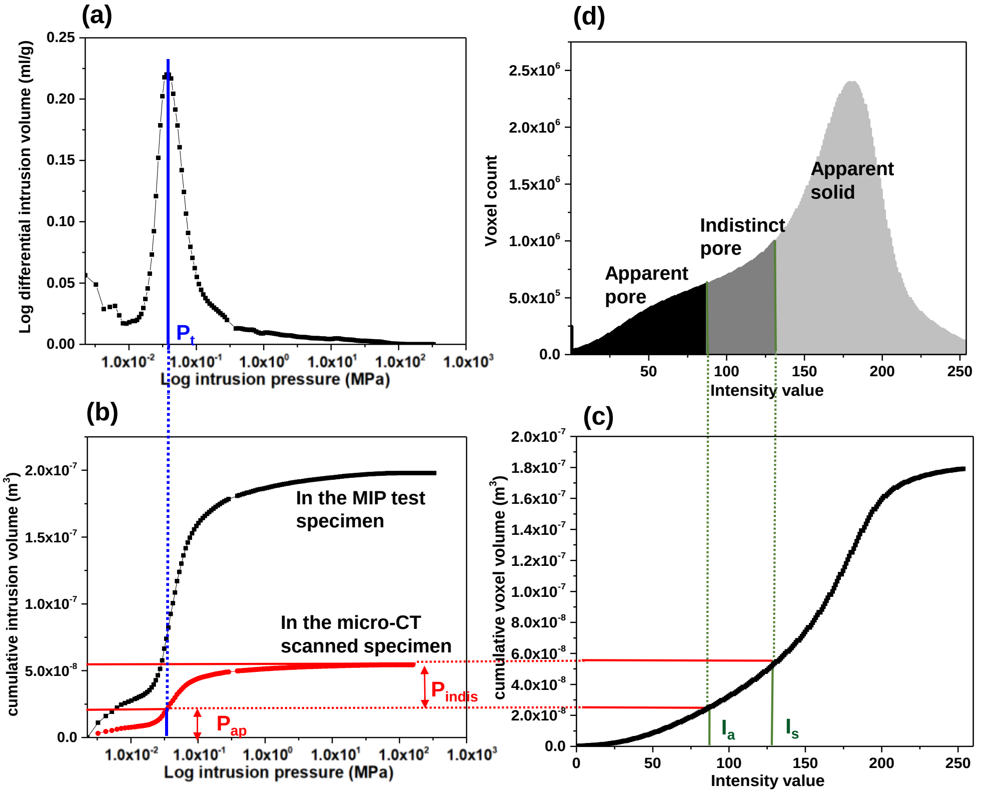

Figure S1. Segmentation and designation processes for three-phase materials consisting of apparent pores (P_{ap}), indistinct pores (P_{indis}) and apparent solid (S_{ap}). Data were obtained from the MIP test (**a**,**b**) and the micro-CT image process on a case of Boise sandstone (**c**,**d**).

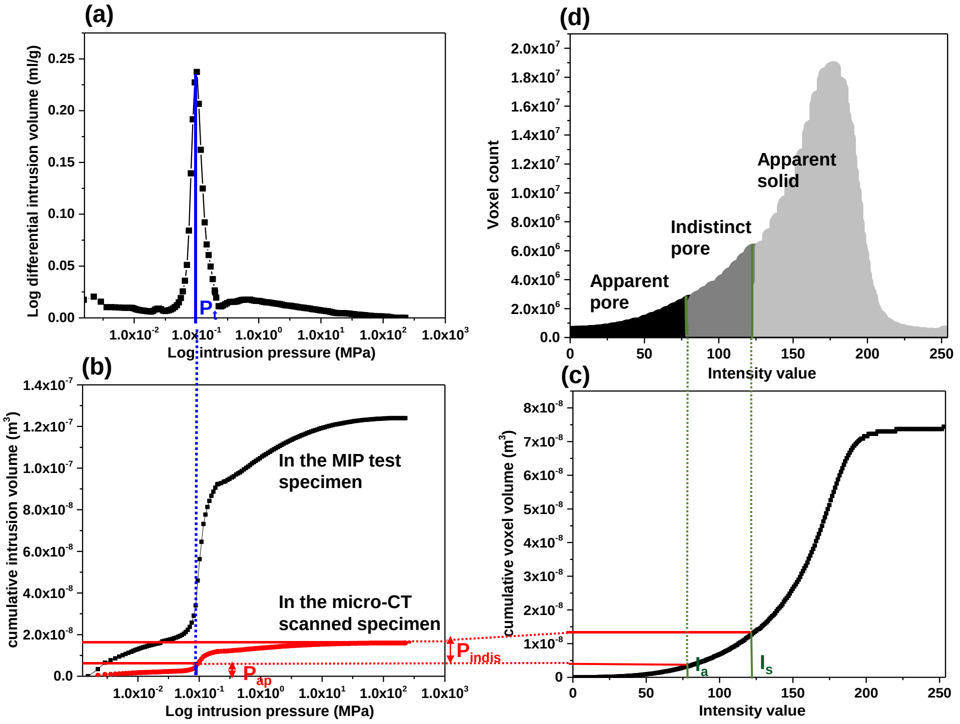

Figure S2. Segmentation and designation processes for three-phase materials consisting of apparent pores (P_{ap}), indistinct pores (P_{indis}) and apparent solid (S_{ap}). Data were obtained from the MIP test (**a**,**b**) and the micro-CT image process on a case of Berea sandstone (**c**,**d**).

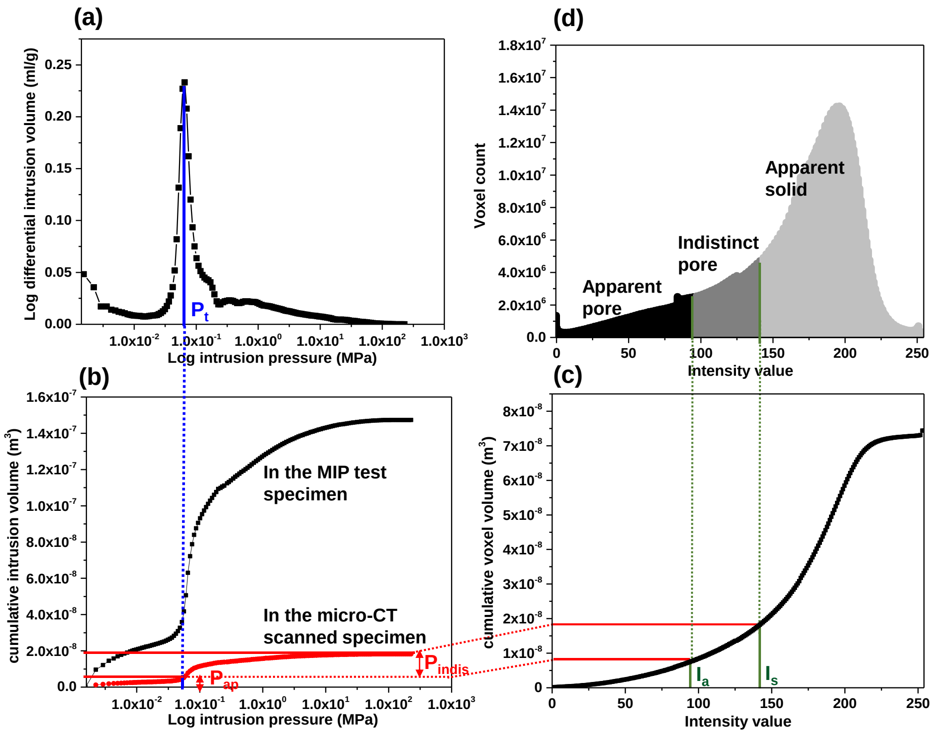

Figure S3. Segmentation and designation processes for three-phase materials consisting of apparent pores (P_{ap}), indistinct pores (P_{indis}) and apparent solid (S_{ap}). Data were obtained from the MIP test (**a**,**b**) and the micro-CT image process on a case of Buff Berea sandstone (**c**,**d**).

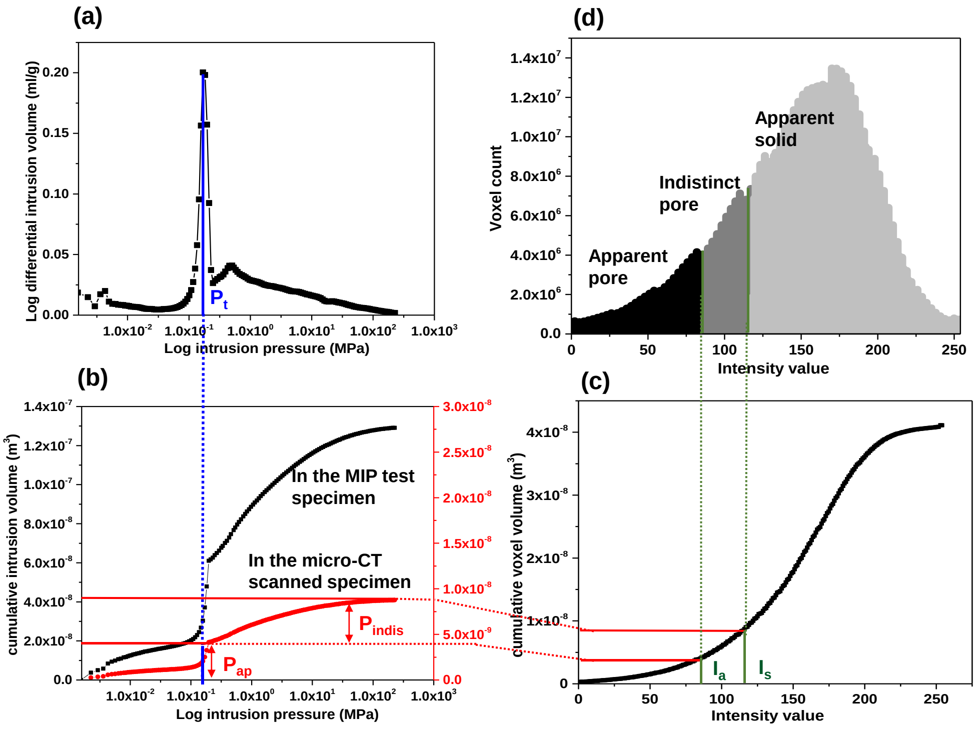

Figure S4. Segmentation and designation processes for three-phase materials consisting of apparent pores (P_{ap}), indistinct pores (P_{indis}) and apparent solid (S_{ap}). Data were obtained from the MIP test (**a**,**b**) and the micro-CT image process on a case of Bandera sandstone (**c**,**d**).

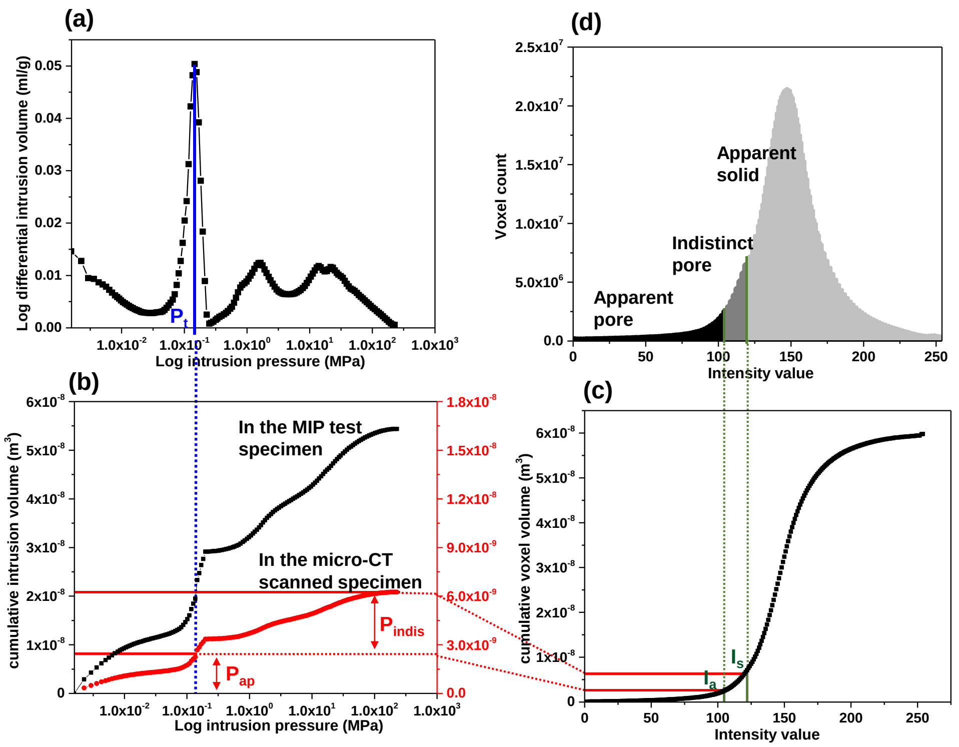

Figure S5. Segmentation and designation processes for three-phase materials consisting of apparent pores (P_{ap}), indistinct pores (P_{indis}) and apparent solid (S_{ap}). Data were obtained from the MIP test (**a**,**b**) and the micro-CT image process on a case of Linyi sandstone (**c**,**d**).

Distinct 3D domains of apparent and indistinct pores (each of P_{ap} and P_{indis}) were constructed through combining a 2D section with representative pore shapes and a 3D tortuous flow path. In the commercial program COMSOL Multiphysics, a coupled flow regime with the two domains was easily meshed and set up. Both the Stokes and Brinkman equations were solved to conduct a fluid flow simulation in the developed pore channel model. The coupling was successfully simulated and evaluated against the experimental results of permeability derived from Darcy's law. For a numerical simulation using the developed pore channel model, an appropriate boundary condition must be assigned to distinguish the pore-scale forces used in the Stokes-Brinkman equation from the core-scale fluid pressure drop found in Darcy flow. To simulate the CO_2 flooding test had been conducted in this laboratory [16], simulation parameters corresponding to the experimental conditions should be imposed on the pore channel model. Table S1 lists the input parameters. For matching the experimental conditions, the CO_2 properties are assumed to be constant across the pore channel model under a temperature of 50 °C and pressure of 10 MPa. Figure S1 compares the core flooding experiment and the numerical simulation based on the developed pore channel model. In accordance with the core flooding test, the superficial velocity was assumed to be a fixed value of about 7.29×10^{-6} m/s to simulate a laminar flow. The upstream condition induced a resistance to fluid flow and gave rise to a pressure increase at the bottom side. Therefore, the permeability of the core specimen and the micro-CT volume fraction could be estimated through the induced pressure gradient along the specimen length of L_s and by considering the distance L_0, which is the voxel height. The *z*-axis straight distance (L_0) on all specimen has a value of about 0.1 mm because of the fixed voxel height in CT image analysis. It should be emphasized that this study considered only one representative pore channel from the micro-CT volume, which had the form of a pore channel bundle. The actual fluid velocity in the connected pores was calculated by considering the existence of a pore throat within the specimen, which accelerated the superficial velocity to preserve fluid continuity. Therefore, the interstitial velocity (actual or pore velocity) in the pore throat simply represents the increased velocity determined by the relation between the superficial velocity and the porosity of the

specimen, as illustrated in Figure S1. In the developed model of a pore channel extracted from the void fraction of the micro-CT volume, the applied inlet flow should take the interstitial velocity at the bottom side of the pore channel. Therefore, each different inlet velocity was assigned according to the porosity of the specimens, as listed in Table S1. After that, the permeability of the micro-CT volume fraction was calculated from the pressure gradient (ΔP_c) along the micro-CT image voxel height (L_o) and the outlet superficial velocity (v_s):

$$K = \frac{\mu v_s L_o}{\Delta P_c}$$

And also, an improvement of the developed pore channel model can be expected by implementing the concept of preferential flow paths. Previous studies showed that fluid flow is most likely to occur along certain preferential flow paths formed in a porous medium. Therefore, firstly, the tortuosity factors (including the tortuosity, average distance and propagation angle of the connected pores) expressed by sinusoidal curve in pore channel model were determined through an investigation of the total pore phase ($P_{ap} + P_{indis}$) for each plane in the stacked image. Secondly, we had attempted to simulate the preferential flow path effect, the tortuosity factors were derived by applying image processing only to P_{ap}. And then we reconstructed a 3D computational domain. The two pore channel model with different tortuous flow path could be used in the COMSOL program. The estimated permeability can be compared through the fluid velocity and pressure field as below tables. As a result, a little more reasonable agreement was found between the permeability derived from the pore channel model with the preferential flow path and the experimentally estimated permeability.

Table S1. Numerical input data corresponding to core flooding experiment using CO_2.

Property	Value		Description
μ	2.842×10^{-5} Pa·s		Dynamic viscosity
v_s	7.29×10^{-6} m/s		Superficial velocity
v_i	Boise	2.51×10^{-5} m/s	Interstitial inlet velocity
	Berea	3.46×10^{-5} m/s	
	Buff Berea	3.08×10^{-5} m/s	
	Bandera	3.56×10^{-5} m/s	
	Linyi	7.35×10^{-5} m/s	
P_0	10 MPa		Initial outlet pressure

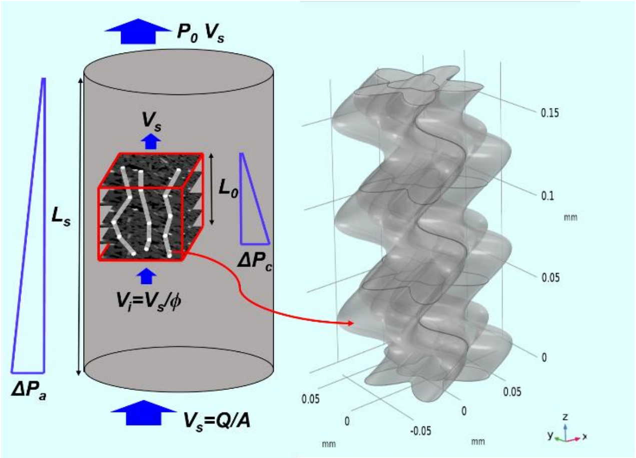

Figure S6. Depiction of fluid pressure and velocity field through a porous specimen and consideration of a micro-CT image volume fraction. Illustration of the experimental condition of steady state flow under a constant pressure gradient. Boundary notation explains the parameters for adopting Darcy's law in both the experiment and numerical simulation.

Table S2. Computational results of fluid pressure and velocity field in the pore channel model of Boise sandstone.

The Total Pore Phase		The Apparent Pore Phase	
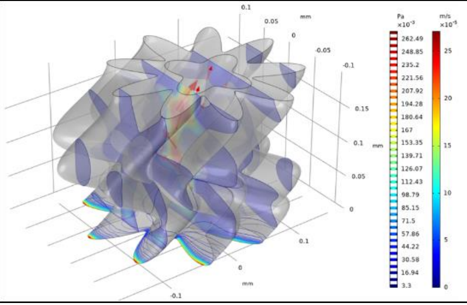		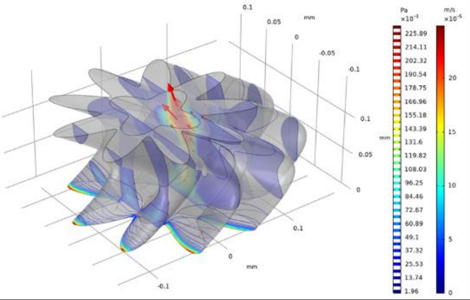	
Average superficial velocity (v_s, 10^{-6}m/s)	7.185	Average superficial velocity (v_s, 10^{-6}m/s)	7.196
Average pressure gradient (ΔP_c, Pa)	0.0216	Average pressure gradient (ΔP_c, Pa)	0.0179

Table S3. Computational results of fluid pressure and velocity field in the pore channel model of Berea sandstone.

The Total Pore Phase.		The Apparent Pore Phase	
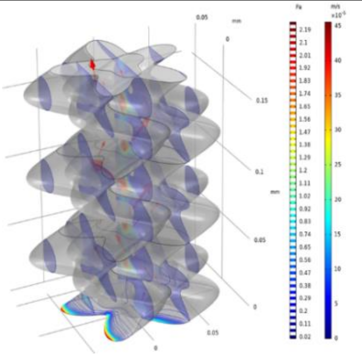		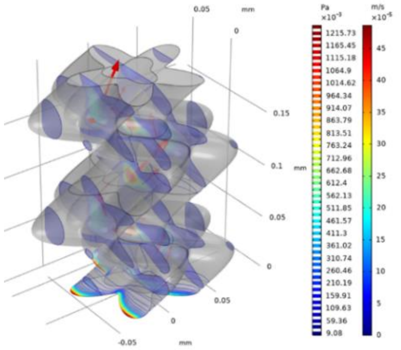	
Average superficial velocity (v_s, 10^{-6}m/s)	7.126	Average superficial velocity (v_s, 10^{-6}m/s)	
Average pressure gradient (ΔP_c, Pa)	0.2016	Average pressure gradient (ΔP_c, Pa)	

Table S4. Computational results of fluid pressure and velocity field in the pore channel model of Buff Berea sandstone.

The Total Pore Phase		The Apparent Pore Phase	
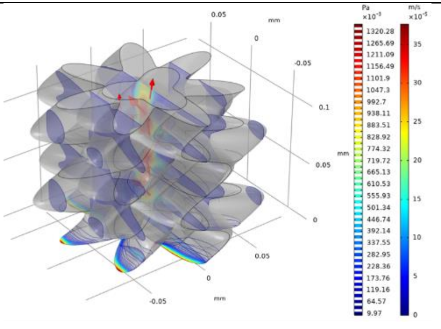		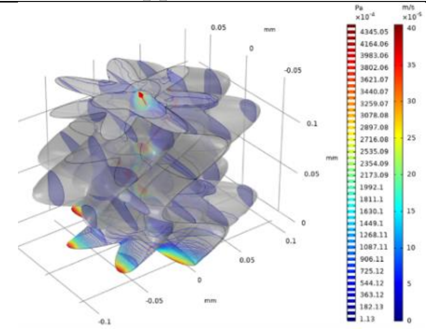	
Average superficial velocity (v_s, 10^{-6}m/s)	7.176	Average superficial velocity (v_s, 10^{-6}m/s)	6.824
Average pressure gradient (ΔP_c, Pa)	0.0939	Average pressure gradient (ΔP_c, Pa)	0.0767

Table S5. Computational results of fluid pressure and velocity field in the pore channel model of Bandera sandstone.

The Total Pore Phase		The Apparent Pore Phase	
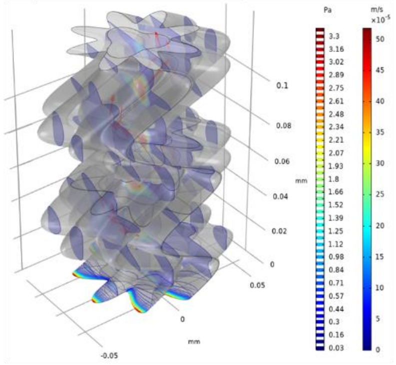		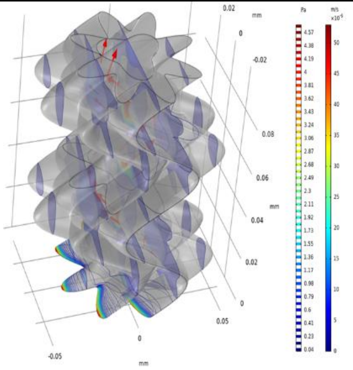	
Average superficial velocity (v_s, 10^{-6}m/s)	7.168	Average superficial velocity (v_s, 10^{-6}m/s)	7.199
Average pressure gradient (ΔP_c, Pa)	0.4064	Average pressure gradient (ΔP_c, Pa)	0.5962

Table S6. Computational results of fluid pressure and velocity field in the pore channel model of Linyi sandstone.

The Total Pore Phase		The Apparent Pore Phase	
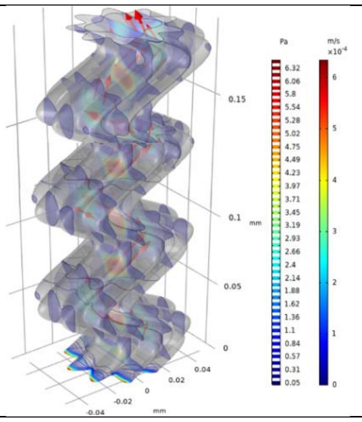		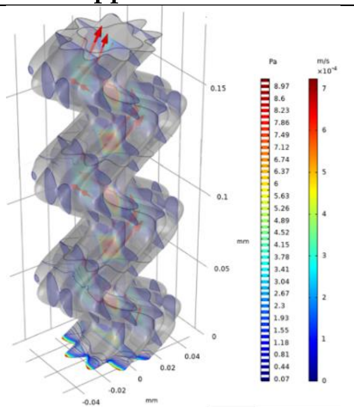	
Average superficial velocity (v_s, 10^{-6}m/s)	7.285	Average superficial velocity (v_s, 10^{-6}m/s)	7.266
Average pressure gradient (ΔP_c, Pa)	1.1496	Average pressure gradient (ΔP_c, Pa)	1.715

The resulting pore geometric description from the image processing could also be utilized for a direct numerical simulation (DNS). We compared the developed pore channel model with DNS in terms of their accuracy and efficiency. In DNS, the velocity and pressure field of the Stokes equation are calculated on a computational mesh representing complex pore spaces based on a discrete approximation. In this study, the simulation was performed using Avizo's XLab module for simulating an absolute permeability experiment based on a finite volume method. To compute a flow field driven by a pressure gradient, it is necessary that the key connected pore structure is identified and modeled to simplify the simulation. The principal pore space being meshed is also dependent on the threshold intensity value of the micro-CT image because the bulk volume size of the pore space is determined by deciding whether or not each voxel is a pore. Thus, same with the simulation of pore channel model, DNS was classified using one of two criteria: the pore space was regarded as either the P_{ap} phase only or as the total pore phase combining the P_{ap} and P_{indis} phases. Application of Darcy's law yields the permeability with fluid velocity and pressure gradient. The fluid viscosity and

boundary condition is same with the COMSOL simulation of the pore channel model. Below figures indicate the results of pressure and velocity fields in all cases.

As a result, reasonable results were acquired when the simulation was performed on the pore structure configured only for the P_{ap}. In the case of the computational domain built from the $P_{ap} + P_{indis}$ phase, an overestimation of large connected pores was caused by considering the P_{indis} in the pore structure as a fully developed flow regime. Therefore, it is suggested that a pore structure constructed solely from P_{ap} should be chosen for DNS.

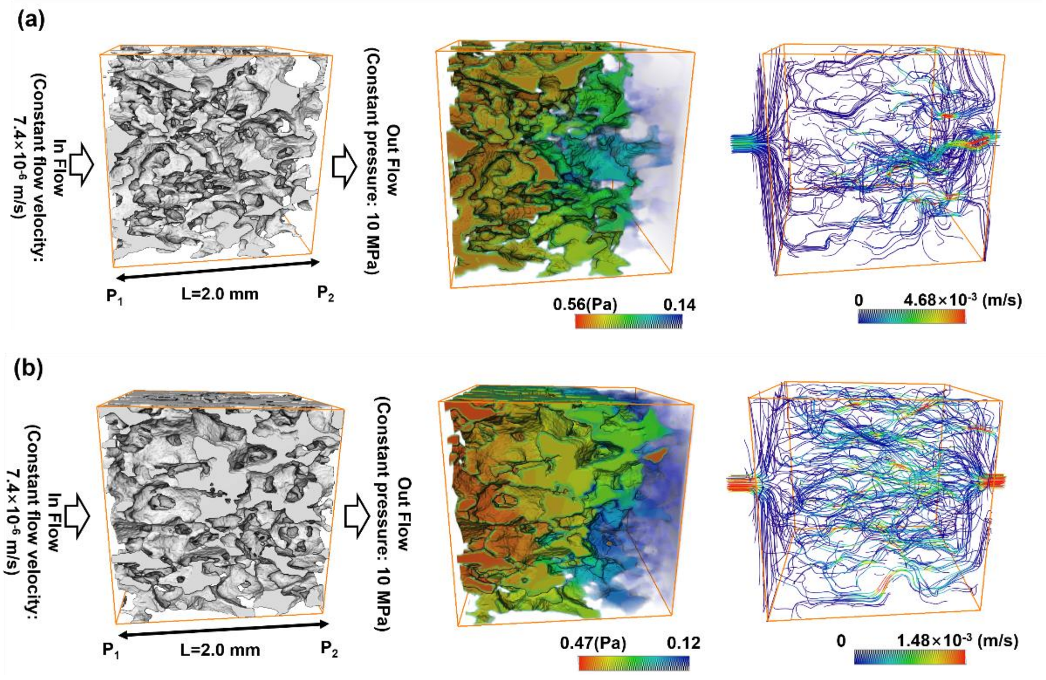

Figure S7. Computed velocity and pressure gradient field dependent on the pore structure in region of interest configured as (**a**) the P_{ap} phase or (**b**) the $P_{ap} + P_{indis}$ phase for Boise sandstone.

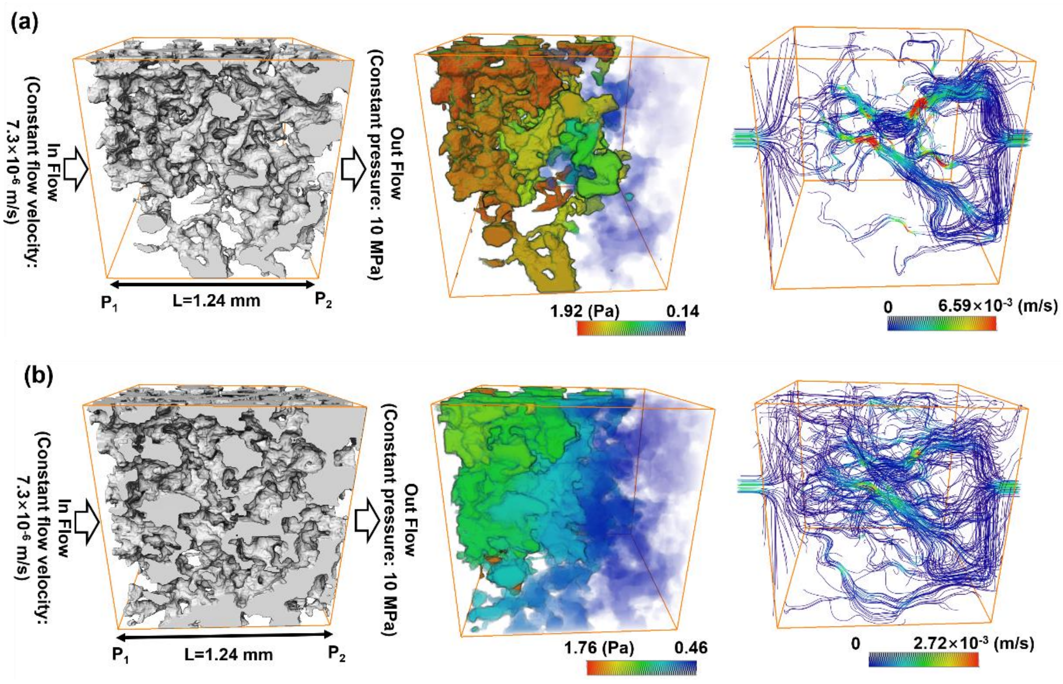

Figure S8. Computed velocity and pressure gradient field dependent on the pore structure in region of interest configured as (**a**) the P_{ap} phase or (**b**) the $P_{ap} + P_{indis}$ phase for Berea sandstone.

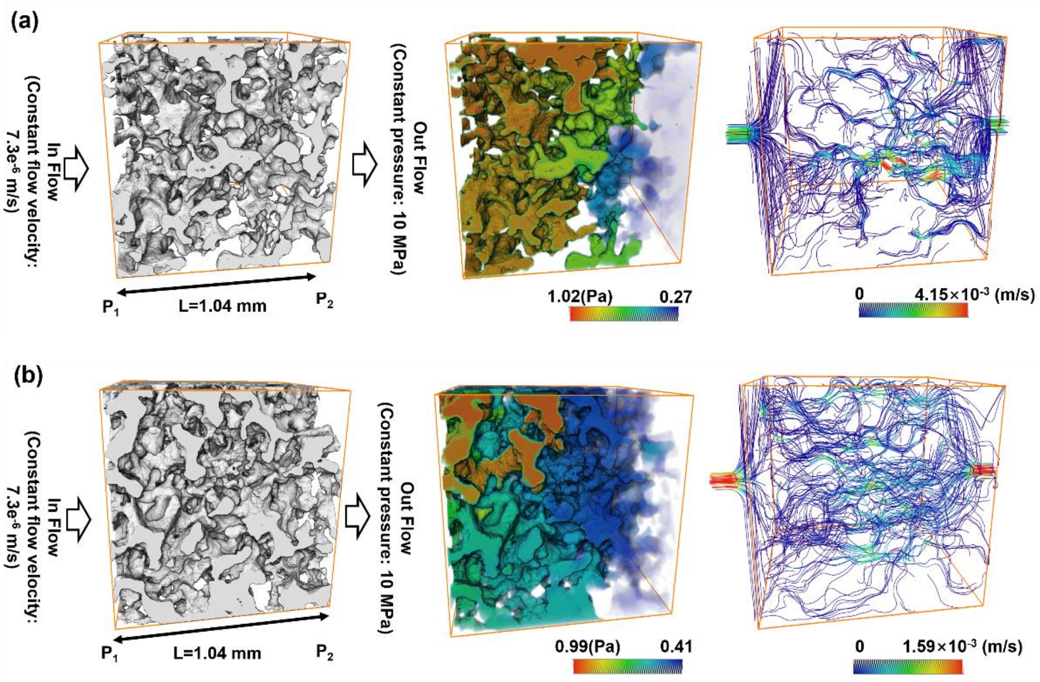

Figure S9. Computed velocity and pressure gradient field dependent on the pore structure in region of interest configured as (**a**) the P_{ap} phase or (**b**) the $P_{ap} + P_{indis}$ phase for Buff Berea sandstone.

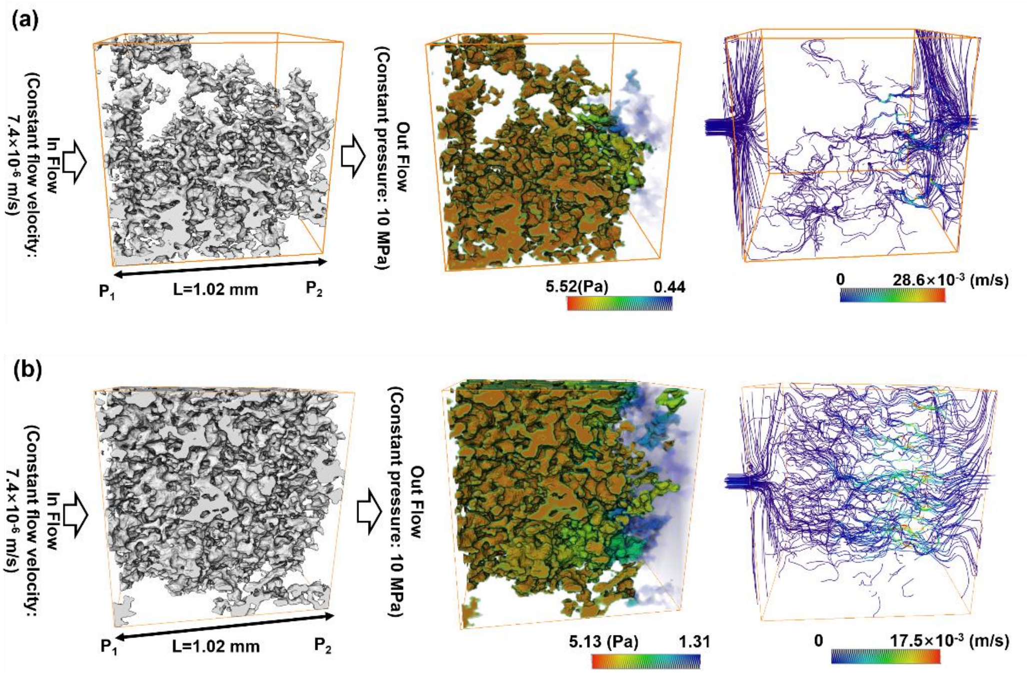

Figure S10. Computed velocity and pressure gradient field dependent on the pore structure in region of interest configured as (**a**) the P_{ap} phase or (**b**) the $P_{ap} + P_{indis}$ phase for Bandera sandstone.

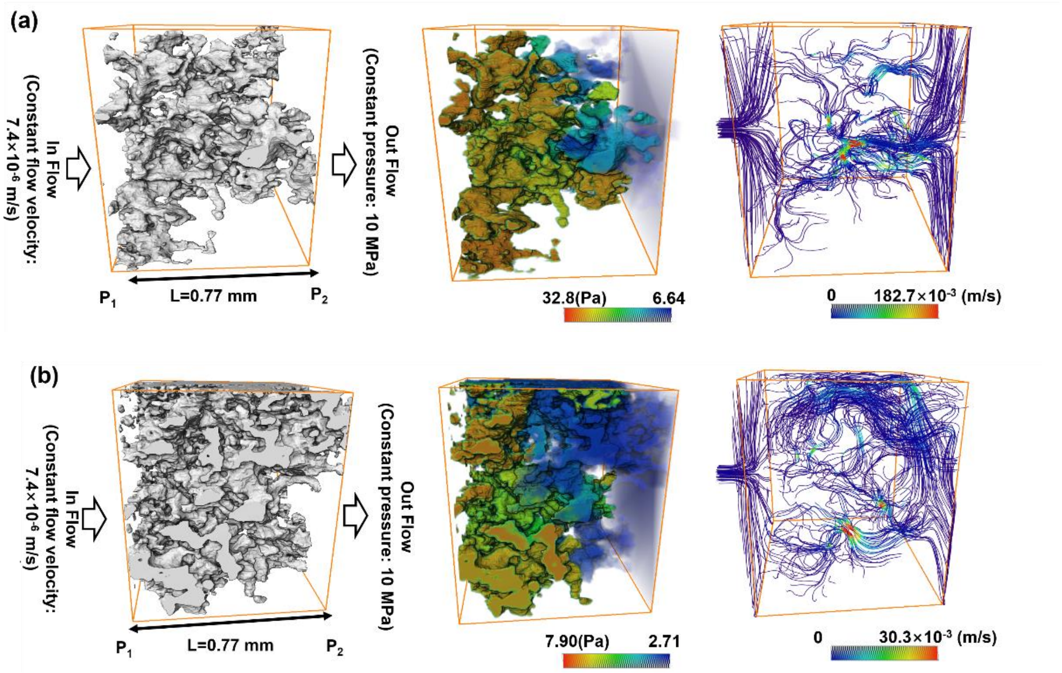

Figure S11. Computed velocity and pressure gradient field dependent on the pore structure in region of interest configured as (**a**) the P_{ap} phase or (**b**) the P_{ap} + P_{indis} phase for Linyi sandstone.
